# Editorial: Focus on maternal mental health during pregnancy and after childbirth

**DOI:** 10.3389/fgwh.2024.1393215

**Published:** 2024-05-06

**Authors:** Margaret I. Fitch, Michaela Hynie

**Affiliations:** ^1^ Bloomberg Faculty of Nursing, University of Toronto, Toronto, ON, Canada; ^2^ Department of Psychology/Centre for Refugee Studies, York University, Toronto, ON, Canada

**Keywords:** maternal mental health, maternal well-being, mental health during pregnancy, maternal depression, maternal anxiety

**Editorial on the Research Topic** Focus on maternal mental health during pregnancy and after childbirth

Maternal mental health disorders are a serious public health problem worldwide. Maternal mental health disorders refer to mental health problems that emerge during pregnancy and/or the year following birth (1). Global incidence of maternal mental health disorders is 10% of women during pregnancy and 13% following childbirth (2). These incidence rates for maternal mental health disorders vary between high income countries (HICs) and low/middle income countries (LMICs). For example, in HICs the incidence of postpartum depression ranges from 5% to 20% while in LMICs, based on limited reliable data available, the incidence ranges from 4.9% to 50% (3). In the case of LMICs, one in four women are reported to develop mental health disorders during pregnancy and one in five following delivery (3).

The most common maternal mental health disorders for the women are anxiety and depression and both may range from mild to moderate or severe. The experience for women often results in not being able to follow through on their responsibilities, reduced work productivity, and decreased ability to cope with the stresses of daily living (4). Evidence shows there is a relationship between antenatal mental health problems on negative birth outcomes, but also that problematic birth outcomes like low birthweight or premature birth are related to subsequent postnatal mental health concerns. Adverse effects on infant cognitive development, nutritional status and early childhood development have been reported (5, 6).

Despite the importance of maternal mental health to the well-being of both mothers and infants, there remains limited evidence to inform policy regarding this serious health issue, especially in LMICs (7). Moreover, although there are programs integrated in HICs at the primary care level for pregnant and postpartum women, the availability of similar programs in LMICs remains limited to non-existent, as the focus is often more on maternal and infant physical care than psychological well-being (8). Often women are not receiving necessary psychological care.

Hence, the topic area of maternal mental health disorders for pregnant and postpartum women in LMICs remains one which is in critical need of research to guide clinical care as well as to inform policy. This special issue was designed to offer researchers from LMICs an opportunity to showcase their current work in the area of mental health for pregnant and postpartum women. We are delighted to have the submissions which you will see published in this special issue. The articles offer insight into examples of current research and offer a call to action with clinical and policy recommendations.

The submissions come from Rwanda (Umuziga et al.), Nigeria (Odufuwa et al.), Ethiopia (Kassaw et al., Tufa et al.), Cameroon (Djatche Miafo et al.), Namibia (Mlambo and Amukugo), India (Mazumdar et al.), and Pakistan (Owais et al.). All are projects led by researchers based in the respective country with two indicting there was an international collaboration on the project. Five are cross-sectional studies (Umuziga et al., Odufuwa et al., Kassaw et al., Tufa et al., Djatche Miafo et al.) while one is a secondary analysis (Owais et al.) of an aspect of an intervention trial and two are qualitative designs (Mlambo and Amukugo, Mazumdar et al.). Three focused on depression (Umuziga et al., Odufuwa et al., Owais et al.) and one on both anxiety and depression (Djatche Miafo et al.). Other foci included mental health disorders (Odufuwa et al., Djatche Miafo et al.), cognitive disorders (Kassaw et al.), fertility desire (Tufa et al.), facilitating childbirth choice (Mlambo and Amukugo) and mindful parenting (Mazumdar et al.). The samples included antenatal (Kassaw et al., Mlambo and Amukugo). postnatal (Umuziga et al., Odufuwa et al., Mazumdar et al., Owais et al.), perinatal (Djatche Miafo et al.), and women living with HIV (Tufa et al.). All but one study used standardized self-report scales while the one utilized a clinically administered measure to diagnose cognitive disorders (Kassaw et al.).

The cross-sectional studies (Umuziga et al., Odufuwa et al., Kassaw et al., Tufa et al., Djatche Miafo et al.) explored predictive factors of high risk for maternal mental health problems or factors associated with the disorders. Two of these collected their data through interviews while the others applied written questionnaires or patient medical chart extraction. The secondary analysis study explored the integration of a mental health intervention in a primary care setting. All quantitative studies employed a systematic sampling technique, described their samples well and utilized standardized measures of selected variables, which aid in being able to make comparisons between settings and over time.

The two qualitative studies (Mlambo and Amukugo, Mazumdar et al.) utilized in-depth interviews followed by a standardized content and thematic analysis. The qualitative studies used purposive sampling to access individuals who had experienced the phenomenon and were willing to talk about their experiences. These types of studies help to gain insight into the culture and practices in the respective countries and are important to tailor future interventions which are tailored to the local context.

Many results were consistent across settings, but some unique factors also emerged. Across the studies, mental disorders were cited at approximately a quarter of the respective samples. Social support emerged as an important factor across multiple studies. Umuziga et al. from Rwanda reported poor perceived health status, poor partner and family support, poor social support, and negative life events (violence) as predictors of depressive symptoms while friend support was a protector. Kassaw et al. from Ethiopia reported strong social support, orthodox religion follower, income level, being over 26 years, unplanned pregnancy, and rural residence dwelling as factors associated with increased cognitive impairments (e.g., orientation, attention, computation, recall, language). Djatche Miafo et al. cited links between anxiety and depression and an absence of social support were factors associated with an increased risk of mental disorders in women from Cameroon. Tufa et al. from Ethiopia described parent and community pressure, being married, having only girls, and having children who are seropositive as factors associated with the desire for fertility in women living with HIV. Desire for fertility is a psychological state whereby women have a motivation for having additional children, yet their HIV status can present challenges and bring about heightened pressures. The findings regarding both support and external pressure from others indicates that attention to mothers' interpersonal relationships is important to understanding and predicting maternal mental health outcomes.

Two studies looked at the delivery of mental health care given women were not having easy access to the needed mental health care. Odufuwa et al. from Nigeria described how both depressed and non-depressed women sought help for maternal mental health care. Age, family history of postpartum depression, currently feeling depressed and having children of the desired gender were associated factors for help seeking. Fear and stigma, thinking the symptoms will go away on their own, and a low perceived need for help held women back from seeking mental health care. The work by Owais et al. emphasized the feasibility of integrating a mental health intervention in a primary health care setting but also revealed gaps in the referral pathway (Owais et al.).

Of the two qualitative studies, one explored the lived experiences of mothering in an urban setting in India (Mazumdar et al.) whereas the other accessed midwives working in Namibia to explore their experiences in facilitating choice by women surrounding childbirth (Mlambo and Amukugo). Both studies emphasized the need for increasing awareness of the issues (e.g., emotional and physical distress, poor social support, lack of time for self, image of self as a mother) and looking at barriers beyond the individual person. For example, facility and health system structural factors (e.g., access to timely care, transportation) also have an impact on the decision-making and actions of mothers.

Across all studies, authors recommended clinical assessment of the mental health status of pregnant and postpartum women. Easily applied and standardized instruments are available for this purpose providing there is review for appropriateness of the tool in the local culture. They also emphasized the need to increase awareness of the reality of these disorders in the general population and for policy changes to facilitate funding to support implementation of mental health care.

The authors also offered clear recommendations regarding the need for further research in this arena. In particular, the development and testing of relevant, locally appropriate, intervention strategies is needed. The need for further description of the incidence, prevalence, risk factors and impact of the disorders within larger populations, and exploration of access to mental health care would be useful.

Overall, these eight articles provide a window into the potential range of work in this topic area and the potential for influencing the well-being of women and their offspring. The papers also underscore the need for action as the impact on mothers and infants is pressing with both immediate and long-term consequences.
